# Supplementary material for: Effective drug combinations in breast, colon and pancreatic cancer cells
Source: Nature. 2022 Feb 23;603(7899):166–73. doi: 10.1038/s41586-022-04437-2 (PMC8891012; doi:10.1038/s41586-022-04437-2)
Supplement: Supplementary file 1 — This file contains images of Western blots described in the paper. [file 41586_2022_4437_MOESM1_ESM.pdf]

nature portfolio

https://doi.org/10.1038/s41586-022-04437-2

Supplementary information

Effective drug combinations in breast, colon and pancreatic cancer cells

In the format provided by the
authors and unedited

Uncropped Western blots of Extended Data Figure 7b

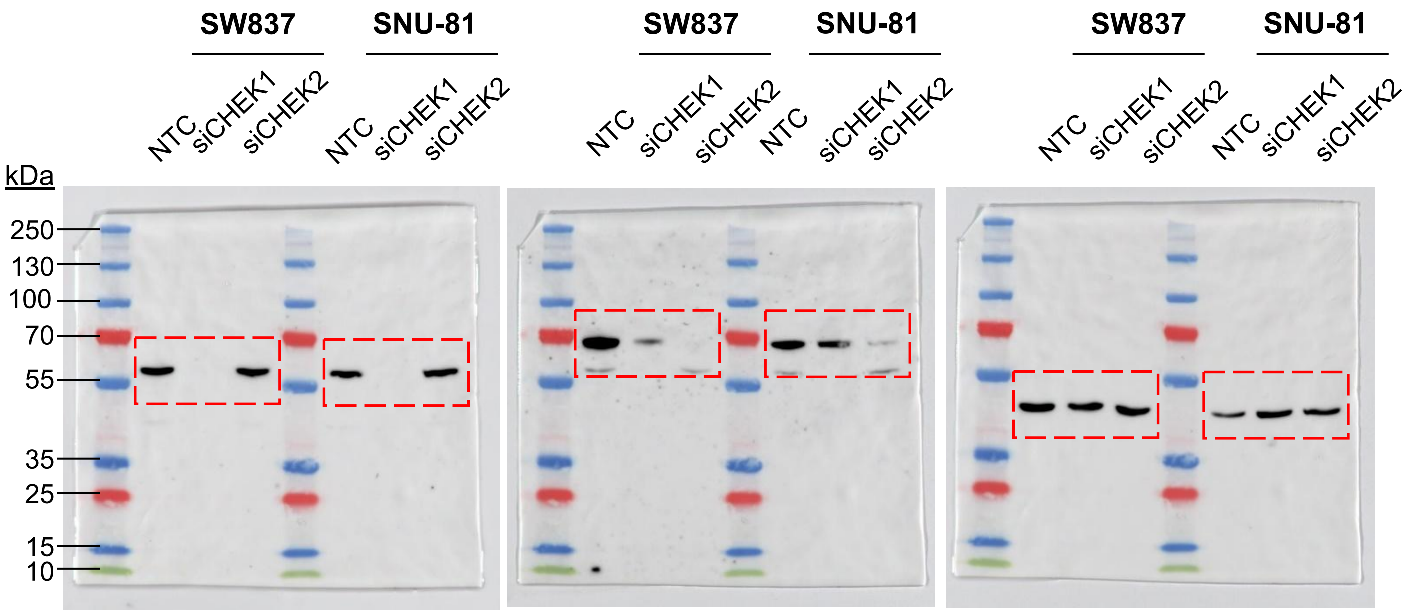

CHEK1: Santa Cruz (sc-8408)
1:500, 5% milk in TBS-T

CHEK2: CST (D9C6)
1:1000, 5% milk in TBS-T

Beta-actin: abcam (ab6276)
1:5000, 5% milk in TBS-T

All three images represent the same gel/membrane. Antibodies were probed one after the other in the following order: CHEK1, CHEK2, actin. We note that upon probing with CHEK2 faint bands of the previously probed CHEK1 are still visible.

Uncropped Western blots of Extended Data Figure 7d

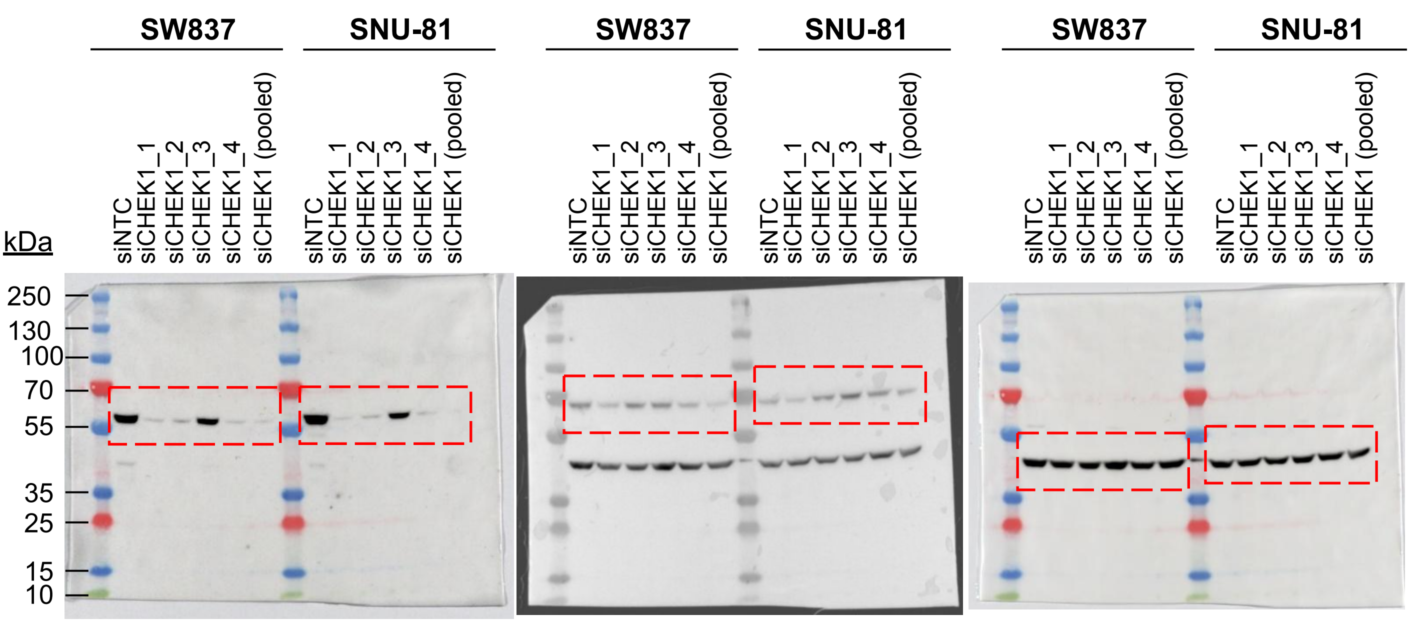

CHEK1: Santa Cruz (sc-8408)
1:500, milk in TBS-T

CHEK2: CST (D9C6)
1:1000, milk in TBS-T

Beta-actin: abcam (ab6276)
1:5000, milk in TBS-T

All three images represent the same gel/membrane. Antibodies were probed one after the other in the following order: CHEK1, actin, CHEK2. We note that upon probing with CHEK2 faint bands of the previously probed actin are still visible.

Area cropped for the figure panel

Uncropped Western blots of Figure 4f

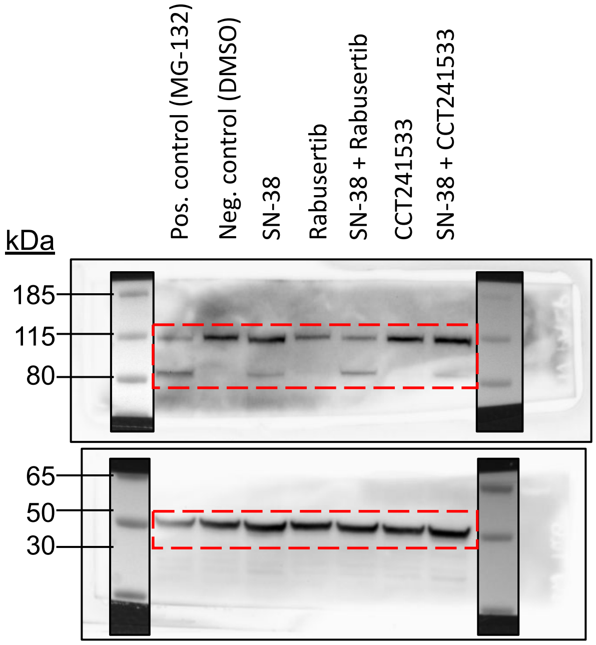

SW837 cells

PARP: Cell Signalling Technologies (9542)
1:1000, 5% milk in TBS-T

PARP at 116 kDa (upper band)
Cleaved PARP at 89 kDa (lower band)

β-tubulin: Sigma-Aldrich (T4026)
1:5000, 5% milk in TBS-T

Tubulin at 55 kDa

The same samples were run on the same gel, transferred and the membrane was cut at the 65 kDa marker band to allow simultaneous probing for PARP and tubulin. Markers were visualised with normal brightfield imaging and pictures were overlaid.

Uncropped Western blots of Extended Data Figure 7i

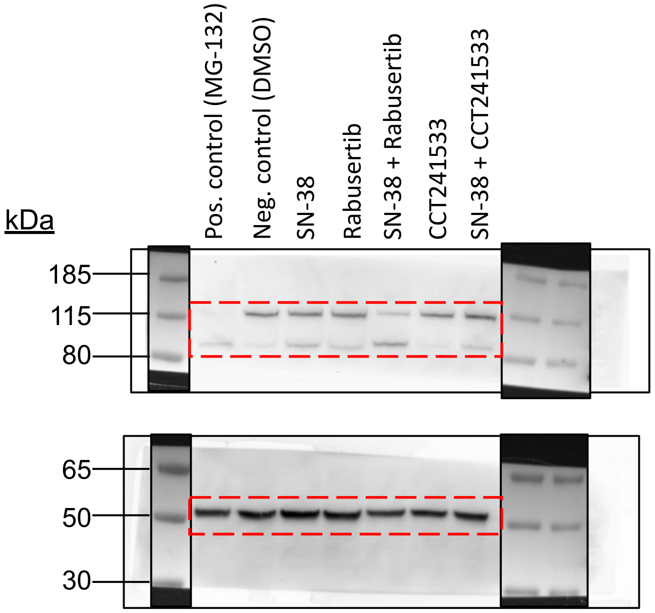

SNU-81 cells

PARP: Cell Signalling Technologies (9542)
1:1000, 5% milk in TBS-T

PARP at 116 kDa (upper band)
Cleaved PARP at 89 kDa (lower band)

β-tubulin: Sigma-Aldrich (T4026)
1:5000, 5% milk in TBS-T

Tubulin at 55 kDa

The same samples were run on the same gel, transferred and the membrane was cut at the 65 kDa marker band to allow simultaneous probing for PARP and tubulin. Markers were visualised with normal brightfield imaging and pictures were overlaid.

Area cropped for the figure panel
