# Supplementary material for: Effective drug combinations in breast, colon and pancreatic cancer cells
Source: Nature. 2022 Feb 23;603(7899):166–73. doi: 10.1038/s41586-022-04437-2 (PMC8891012; doi:10.1038/s41586-022-04437-2)
Supplement: Supplementary file 2 — Reporting Summary [file 41586_2022_4437_MOESM2_ESM.pdf]

nature portfolio

Corresponding author(s): Garnett, Mathew

Last updated by author(s): Dec 21, 2021

Reporting Summary

Nature Portfolio wishes to improve the reproducibility of the work that we publish. This form provides structure for consistency and transparency in reporting. For further information on Nature Portfolio policies, see our Editorial Policies and the Editorial Policy Checklist.

Statistics

For all statistical analyses, confirm that the following items are present in the figure legend, table legend, main text, or Methods section.

n/a	Confirmed	
☐	☒	The exact sample size (n) for each experimental group/condition, given as a discrete number and unit of measurement
☐	☒	A statement on whether measurements were taken from distinct samples or whether the same sample was measured repeatedly
☐	☒	The statistical test(s) used AND whether they are one- or two-sided<br>*Only common tests should be described solely by name; describe more complex techniques in the Methods section.*
☐	☒	A description of all covariates tested
☐	☒	A description of any assumptions or corrections, such as tests of normality and adjustment for multiple comparisons
☐	☒	A full description of the statistical parameters including central tendency (e.g. means) or other basic estimates (e.g. regression coefficient) AND variation (e.g. standard deviation) or associated estimates of uncertainty (e.g. confidence intervals)
☐	☒	For null hypothesis testing, the test statistic (e.g. F, t, r) with confidence intervals, effect sizes, degrees of freedom and P value noted<br>*Give P values as exact values whenever suitable.*
☒	☐	For Bayesian analysis, information on the choice of priors and Markov chain Monte Carlo settings
☒	☐	For hierarchical and complex designs, identification of the appropriate level for tests and full reporting of outcomes
☐	☒	Estimates of effect sizes (e.g. Cohen's d, Pearson's r), indicating how they were calculated

Our web collection on statistics for biologists contains articles on many of the points above.

Software and code

Policy information about availability of computer code

Data collection	no software was used
Data analysis	The software package GDSC Tools v1.0.1 was used for biomarker analysis (https://doi.org/10.1093/bioinformatics/btx744). Dose response curves were fitted using a 2-parameter sigmoid function (https://doi.org/10.2217/pgs.16.15)

For manuscripts utilizing custom algorithms or software that are central to the research but not yet described in published literature, software must be made available to editors and reviewers. We strongly encourage code deposition in a community repository (e.g. GitHub). See the Nature Portfolio guidelines for submitting code & software for further information.

Data

Policy information about availability of data

All manuscripts must include a data availability statement. This statement should provide the following information, where applicable:

- Accession codes, unique identifiers, or web links for publicly available datasets
- A description of any restrictions on data availability
- For clinical datasets or third party data, please ensure that the statement adheres to our policy

All drug sensitivity data generated or analysed during this study are included in this published article (and its supplementary information files) or available in Figshare repository and GDSC Combinations database (https://gdsc-combinations.depmap.sanger.ac.uk/). Cell line metadata and genomic datasets are available from the Cell Model Passports database (https://doi.org/10.1093/nar/gky872). Users have a non-exclusive, non-transferable right to use data files for internal proprietary research and educational purposes, including target, biomarker and drug discovery. Excluded from this licence are use of the data (in whole or any significant part) for resale either alone or in combination with additional data/product offerings, or for provision of commercial services.

Field-specific reporting

Please select the one below that is the best fit for your research. If you are not sure, read the appropriate sections before making your selection.

☒ Life sciences ☐ Behavioural & social sciences ☐ Ecological, evolutionary & environmental sciences

For a reference copy of the document with all sections, see nature.com/documents/nr-reporting-summary-flat.pdf

Life sciences study design

All studies must disclose on these points even when the disclosure is negative.

Sample size: For drug sensitivity testing and biomarker analyses we used all available cancer cell lines available.

Data exclusions: There were no data exclusions

Replication: To assess the reproducibility within a screen, we generated 2-18 biological replicates for 4-5 cell lines per tissue (breast: 5 (AU565, BT-474, CAL-85-1, HCC1937, MFM-223); colon: 4 (HCT-15, HT-29, SK-CO-1, SW620); pancreas: 5 (KP-1N, KP-4, MZ1-PC, PA-TU-8988T, SUIT-2)). Single-agent and combination responses were averaged across technical replicates (typically three per biological replicate) and correlated (Pearson correlation coefficient; minimum of 322 biological replicate pairs per 'metric-tissue' pair).

To assess the reproducibility of the screen, we rescreened a subset of combinations in each tissue (breast: 51 combos in 34 cell lines; colon: 45 combos in 37 cell lines; pancreas: 59 combos in 29 cell lines; Supplementary table 2). Drug combination responses were averaged across replicates within a screen and key metrics of single-agent and combination response were correlated between the two screens (Pearson correlation coefficient). To determine the quality of synergy calls, the original screen was considered as ground truth and numbers of true positive (TP), false positive (FP), true negative (TN) and false negative (FN) synergistic combination-cell line pairs were calculated. These were used to calculate F-score (F-score =TP / (TP + 0.5*(FP + FN))), recall (recall = TP / (TP + FN)), and precision (precision = TP / (TP + FP)) per tissue. To investigate the strength of effects of ΔEmax and ΔIC50 of FP and FN measurements, the distance to ΔEmax and ΔIC50 synergy thresholds was calculated for each 'anchor concentration-library-cell line' tuple based on combination responses averaged across replicates (n=9,570 tuples).

Randomization: For drug sensitivity testing and biomarker analysis, cell lines were organized into groups based on their their tissue and the presence of specific driver mutations or gene expression signatures.

Blinding: Blinding was not required as we used an unsupervised approach for biomarker analysis within defined cohorts.

Reporting for specific materials, systems and methods

We require information from authors about some types of materials, experimental systems and methods used in many studies. Here, indicate whether each material, system or method listed is relevant to your study. If you are not sure if a list item applies to your research, read the appropriate section before selecting a response.

Materials & experimental systems

n/a	Involved in the study
☐	☒ Antibodies
☐	☒ Eukaryotic cell lines
☒	☐ Palaeontology and archaeology
☐	☒ Animals and other organisms
☒	☐ Human research participants
☒	☐ Clinical data
☒	☐ Dual use research of concern

Methods

n/a	Involved in the study
☒	☐ ChIP-seq
☒	☐ Flow cytometry
☒	☐ MRI-based neuroimaging

Antibodies

Antibodies used: The following primary antibodies were used for immunoblot analysis: anti-PARP (Cell Signalling Technologies, 9542, 1:1,000; rabbit), anti-CHEK1 (Santa Cruz Biotechnology, sc-8408, 1:200; mouse), anti-CHEK2 (Cell Signaling Technologies, D9C6, 1:1000; rabbit), anti-β-tubulin (Sigma-Aldrich, T4026, 1:5,000; mouse) as loading control. Anti-Mouse IgG (GE Healthcare, #NA931) and anti-rabbit (GE Healthcare, #NA934) HRP-linked secondary antibodies were used as secondary antibodies. For immunohistochemical analysis with the following antibodies: anti-Ki-67(MIB-1)(Dako #GA626, 1:100; mouse), anti-cleaved caspase-3 (Asp175)(Cell Signaling #9661, 1:200; rabbit) and anti-phospho-histone H2AX (Ser139)(20E3)(Cell Signaling #9718, 1:400; rabbit).

Validation: All antibodies were validated by commercial vendors for Western blotting or immunohistochemistry, as required. anti-CHEK1 and anti-CHEK2 antibodies were validated using siRNA.

Eukaryotic cell lines

Policy information about cell lines

Cell line source(s)	Cell lines were sourced from commercial vendors. Further information on the cell lines used in this study, including their source and molecular profiling datasets can be found on cellmodelpassports.sanger.ac.uk and in Supplementary Table 2. Cell lines used in this study were: AU565, BT-20, BT-474, BT-483, BT-549, CAL-120, CAL-148, CAL-51, CAL-85-1, CAMA-1, COLO-824, DU-4475, EFM-19, EFM-192A, EVSA-T, HCC1143, HCC1187, HCC1395, HCC1419, HCC1428, HCC1500,HCC1569, HCC1599, HCC1806, HCC1937, HCC1954, HCC202, HCC2157, HCC2218, HCC38, HCC70, HDQ-P1, Hs-578-T, JIMT-1, MCF7, MDA-MB-157, MDA-MB-175-VII, MDA-MB-231, MDA-MB-330, MDA-MB-361, MDA-MB-415, MDA-MB-436, MDA-MB-453, MDA-MB-468, MFM-223, MRK-nu-1, OCUB-M, T47D, UACC-812, UACC-893, ZR-75-30, C2BBe1, CaR-1, CCK-81, CL-11, COLO-205, COLO-320-HSR, COLO-678, CW-2, DiFi, GP5d, HCC2998, HCT-116, HCT-15, HT-115, HT-29, HT55, KM12, LoVo, LS-1034, LS-123, LS-180, LS-411N, LS-513, MDST8, NCI-H508, NCI-H716, NCI-H747, RCM-1, RKO, SK-CO-1, SNU-1040, SNU-175, SNU-407, SNU-81, SNU-C1, SNU-C2B, SNU-C5, SW1116, SW1417, SW1463, SW48, SW620, SW837, SW948, T84, AsPC-1, BxPC-3, CAPAN-1, CAPAN-2, CFPAC-1, DAN-G, HPAC, Hs-766T, HuP-T3, HuP-T4, KP-1N, KP-2, KP-3, KP-4, MIA-PaCa-2, MZ1-PC, PA-TU-8902, PA-TU-8988T, PANC-02-03, PANC-03-27, PANC-04-03, PANC-08-13, PANC-10-05, PL4, PSN1, SU8686, SUIT-2, SW1990, YAPC.
Authentication	To prevent cross-contamination or misidentification, all cell lines were profiled using a panel of 94 SNPs (Fluidigm, 96.96 Dynamic Array IFC). Short tandem repeat (STR) analysis was also performed, and cell line profiles were matched to those generated by the cell line repository.
Mycoplasma contamination	All cell lines are routinely tested for mycoplasma and are negative for mycoplasma
Commonly misidentified lines (See ICLAC register)	All cell lines have been manually curated to remove any commonly misidentified cell lines, and SNP and STR authenticated to ensure their identity.

Animals and other organisms

Policy information about studies involving animals; ARRIVE guidelines recommended for reporting animal research

Laboratory animals	NOD/SCID mice
Wild animals	*Provide details on animals observed in or captured in the field; report species, sex and age where possible. Describe how animals were caught and transported and what happened to captive animals after the study (if killed, explain why and describe method; if released, say where and when) OR state that the study did not involve wild animals.*
Field-collected samples	*For laboratory work with field-collected samples, describe all relevant parameters such as housing, maintenance, temperature, photoperiod and end-of-experiment protocol OR state that the study did not involve samples collected from the field.*
Ethics oversight	Animal procedures were approved by the Italian Ministry of Health (authorization 806/2016-PR).

Note that full information on the approval of the study protocol must also be provided in the manuscript.
